# Advances in the Role of the CD40–CD154 Pathway in the Pathogenesis of Diabetic Retinopathy

**DOI:** 10.3390/ijms27093862

**Published:** 2026-04-27

**Authors:** Carlos S. Subauste

**Affiliations:** 1 Division of Infectious Diseases and HIV Medicine, Department of Medicine, Case Western Reserve University, Cleveland, OH 44106, USA; carlos.subauste@case.edu; Tel.: +1-216-368-2785; Fax: +1-216-369-2034; 2 Department of Ophthalmology, Case Western Reserve University, Cleveland, OH 44106, USA; 3 Department of Pathology, Case Western Reserve University, Cleveland, OH 44106, USA; 4 Department of Pharmacology, Case Western Reserve University, Cleveland, OH 44106, USA

**Keywords:** inflammation, cytokines, VEGF, Müller cells, endothelial cells

## Abstract

Diabetic retinopathy is one of the most important complications of diabetes. It is a leading cause of visual loss in the world. While adequate control of hyperglycemia, hypertension and hyperlipidemia can decrease the prevalence of diabetic retinopathy, 60% of patients with type 2 diabetes develop this complication whereas it is estimated that most patients with type 1 diabetes will develop diabetic retinopathy. Upregulation of various pro-inflammatory molecules and vascular endothelial growth factor (VEGF) play a central role in the pathogenesis of the disease. Here we review the role of CD40 as an upstream inducer of these abnormalities and the development of diabetic retinopathy.

## 1. Diabetic Retinopathy

Diabetic retinopathy (DR) is a disease driven by hyperglycemia-induced metabolic abnormalities that include activation of the protein kinase C (PKC) pathway, increased flux through the polyol pathway, activation of the hexosamine pathway and formation of advanced glycation end products (AGEs) [1]. These metabolic derangements are considered to promote low-level chronic inflammation. The upregulation of multiple pro-inflammatory molecules in the retina is central to the pathogenesis of DR. Moreover, increased expression of VEGF is a key driver of retinal vascular leakage and macular edema as well as angiogenesis and the development of proliferative diabetic retinopathy (PDR). While significant advances have been made in the field, the precise molecular events that promote the upregulation of pro-inflammatory molecules and VEGF are still incompletely understood. Given the multiple mediators that are involved in the pathogenesis of DR and the lack of optimal treatment against this disease, studies aimed at the identification of upstream inducers of pathogenic events in DR may lead to effective approaches to treat this disease. This review focuses on the role of the CD40–CD154 pathway in the pathogenesis of DR and includes a discussion of the potential translational application of approaches to block CD40 signaling for the treatment of DR.

## 2. Chronic Inflammation in DR

Chronic inflammation plays an important role in the pathogenesis of DR [2,3,4,5]. This assertion is supported by the increased expression of various pro-inflammatory molecules in animals and patients with DR and the protective effects against DR induced by genetic or pharmacologic disruption of inflammatory mediators or the use of global anti-inflammatory agents in animals [2,3,4]. 

The expression of ICAM-1 is increased in retinal capillaries from diabetic animals and patients with DR [6,7]. Moreover, leukocytes from diabetic animals exhibit increased expression of CD18, the β chain of β_2_-integrins that functions as ICAM-1 ligand [8]. The resulting increase in adherence of leukocytes to retinal capillaries (leukostasis) would lead to capillary obstruction, disruption of the blood–retinal barrier, and endothelial and pericyte cell death leading to the formation of degenerate capillaries [6,9,10,11,12]. The development of the latter structures is important because degenerate capillaries lack blood flow and promote retinal ischemia. Mice deficient in ICAM-1 or CD18 or treated with antibodies against ICAM-1 or CD18 are protected from leukostasis and vascular leakage, as well as the loss of endothelial cells and pericytes and the formation of degenerate capillaries [8,9,10,13].

There is an influx of monocytes into the diabetic retina that is promoted by the chemokine CCL2 [11]. The expression of CCL2 is increased in the retina of diabetic mice [14] as well as in endothelial cells and myofibroblast in epiretinal membranes from patients with PDR [15]. The upregulation of CCL2 in the retina of diabetic mice is accompanied by increased infiltration by CX3CR1^+^/CD11b^+^ monocyte/macrophages [14]. Diabetic *Ccl2^-/-^* mice are not only protected from monocyte/macrophage infiltration but also from vascular leakage [14]. Moreover, administration of a dual CCR2/CCR5 inhibitor (TAK-779) to diabetic mice reduced macrophage/microglial infiltration and vascular leakage, further supporting the importance of CCL2 in the pathogenesis of DR [16].

Pro-inflammatory cytokines play an important role in the development of DR. TNF-α expression is increased in the retina of diabetic rats [17], retinal microglia/macrophages from diabetic mice [18] as well as in endothelial cells and stromal cells in epiretinal membranes from patients with PDR [19]. This cytokine promotes ICAM-1 upregulation, leukostasis, breakdown of the blood–retinal barrier and capillary degeneration [17,20]. These effects were reduced by administration of a soluble tumor necrosis factor α (TNF-α) receptor/Fc construct (TNFR-Fc, etanercept) that inhibits TNF-α signaling [17]. IL-1β and caspase-1, the enzyme that produces IL-1β, play an important role in diabetes-induced retinal pathology. The expression of caspase-1 and IL-1β are increased in the retinas of diabetic mice [21]. This cytokine promotes capillary degeneration in diabetic animals [21]. Moreover, the administration of minocycline (caspase-1 inhibitor) to diabetic animals or genetic deletion of IL-1 prevented the development of capillary degeneration [21].

Inducible nitric oxide 2 (NOS2) and cyclooxygenase 2 (COX-2) also promote the development of DR. The expression of NOS2 is increased in the retinas of diabetic rodents and of patients with DR [22,23]. In addition, diabetic *Nos2^-/-^* mice are protected from leukostasis, superoxide generation and capillary degeneration [24,25]. Diabetic rats exhibit increased expression of COX-2 as well and of prostaglandin E2 [26], and COX-2 is expressed in endothelial and stromal cells in epiretinal membranes from patients with PDR [27].

Studies in vitreal samples from patients with DR revealed elevated concentrations of ICAM-1, TNF-α, IL-1β, IL-6, IL-8 and CCL2 as well as an association between the levels of pro-inflammatory molecules and the severity of disease [28,29,30,31,32,33]. These findings together with the studies in animal models of DR discussed above and the report that achieving good glycemic control in diabetic rats fails to reverse the upregulation of TNF-α, IL-1β, ICAM-1, VCAM-1 and NOS2 [34] provide rationale for the use of pharmacologic inhibition of pro-inflammatory molecules as a therapeutic approach in patients with DR. While corticosteroids reduce expression of pro-inflammatory molecules and are effective against DR, their use carries significant side-effects [35]. Studies on the use of biologics that target specific pro-inflammatory molecules for the treatment of DR have yet to reveal conclusive evidence of their efficacy [36,37]. Given that multiple inflammatory molecules are involved in the pathogenesis of DR, it could be argued that aiming at a single target may not be the most effective therapeutic approach. Therefore, the identification of upstream inducers of multiple pro-inflammatory responses may facilitate the identification of effective strategies to treat DR. 

## 3. Expression of CD40 in the Diabetic Retina

CD40 has been identified as a molecule that triggers upregulation of pro-inflammatory molecules in DR. CD40 is a TNF receptor superfamily member that is expressed in various hematopoietic cells including B cells, dendritic cells, microglia and macrophages [38]. Under non-inflammatory conditions, non-hematopoietic cells either lack detectable expression of CD40 or express this receptor at low levels [39,40]. 

**Figure 1:**
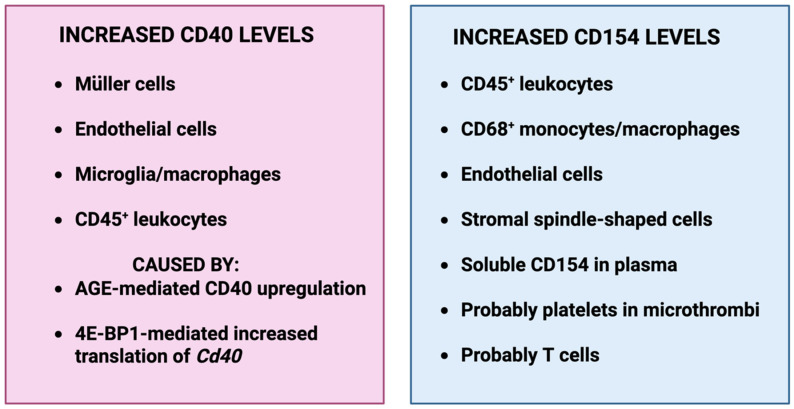
The expression of CD40 and CD154 is increased in DR. Created with BioRender.com.

CD40 is normally expressed at low levels in retinal endothelial cells, Müller cells, microglia and ganglion cells in both retinas from mice and humans [41,42,43]. CD40 is either absent or weakly expressed in human retinal pigment epithelial cells but can be upregulated by IFN-γ [44,45]. In contrast, photoreceptors lack CD40 expression [42]. The expression of CD40 is increased in endothelial cells, Müller cells and microglia from diabetic mice [41] (Figure 1). Studies in patients with diabetic retinopathy also reported upregulation of CD40 in endothelial cells, Müller cells and cells that are likely microglia/macrophages [43]. Moreover, CD40 levels in vitreous samples from PDR patients were significantly higher than the levels in nondiabetic controls [46]. In addition, epiretinal membranes in patients with PDR revealed increased CD40 expression in endothelial cells, CD45^+^ leukocytes, and CD68^+^ monocytes/macrophages [46] (Figure 1). These findings are important since CD40 upregulation is a hallmark of diseases driven by CD40 [40,47]. 

Advanced glycation end products (AGEs) are implicated in complications of diabetes including DR [48]. Indeed, proteins that contain AGE-induced modifications have been associated with upregulation of CD40 in the retinas of patients with DR [49]. Fibronectin and laminin that express the AGE modification carboxymethyl lysine (CML) accumulate in the retinas of patients with DR, and the areas of increased CML expression associated with areas of CD40 upregulation in Müller and endothelial cells [49]. Moreover, in vitro studies revealed that proteins modified by AGEs upregulate CD40 in human retinal endothelial and Müller cells, as well as in monocytes supporting a direct association between AGEs and CD40 upregulation in DR [49,50] (Figure 1). The engagement of AGEs to their receptors increases transcription of numerous pro-inflammatory molecules. It remains to be determined whether this is the mechanism by which AGEs upregulate CD40. Another study supported that translational regulation of *Cd40* mRNA contributes to increased CD40 protein expression in DR [51]. The post-translational protein modification by the O-linked addition of β-D-GlcNAc (O-GlcNAcylation) that is observed in DR was associated with increased translation of *Cd40* mRNA in Müller cells, an effect that required the presence of the translational regulator 4E-BP1 [51] (Figure 1). Taken together, there may be both transcriptional and translational mechanisms that explain CD40 upregulation in DR. 

## 4. Expression of CD154 in the Diabetic Retina

CD154 (CD40 ligand) is a member of the TNF superfamily that is expressed mainly on activated CD4^+^ T cells and platelets, but also as a biologically active soluble protein in plasma [38]. CD154 can also be expressed in various hematopoietic and non-hematopoietic cells supporting the broad role of the CD40–CD154 in biology [52]. CD154 is also upregulated in diabetic retinopathy. The vitreous of patients with PDR exhibit increased expression of CD154 [46,53]. Interestingly, increased vitreal levels of CD154 in patients with PDR were elevated in a manner that was statistically independent of VEGF, suggesting that CD154 has disease-predictive ability independent of VEGF [53]. Endothelial cells, CD45^+^ leukocytes, CD68^+^ monocytes/macrophages and stromal spindle-shaped cells (likely myofibroblasts) are reported to exhibit increased expression of CD154 [46] (Figure 1). Given that CD154 present on activated platelets triggers pro-inflammatory responses in endothelial cells [54], the microthrombi present in the retinal capillaries of patients and animals with DR [55] may provide an additional source of CD154. In addition, T cells that infiltrate the intraocular space in DR may represent an additional source of CD154 [56]. Thus, there appear to be multiple intraocular sources of CD154 that may activate CD40 signaling in DR. Finally, diabetic mice [57] and patients with diabetes, particularly those with microangiopathy, exhibit increased plasma levels of functional CD154 [58,59,60,61,62]. Taken together, there is ample evidence for the activation of the CD40–CD154 pathway in DR (Figure 1).

## 5. CD40 Upregulates Pro-Inflammatory Molecule Expression in Retinal Cells and Is Required for the Development of Experimental DR

CD40 stimulation upregulates a broad range of pro-inflammatory molecules relevant to DR. CD40 stimulation of retinal endothelial cells increases the expression of ICAM-1 and CCL2 [63]. In the case of Müller cells CD40 stimulation upregulates CCL2, ICAM-1, PGE_2_ and the pro-inflammatory alarmin HGMB1 [46,63]. Of relevance to DR, the increased expression of CD40 induced by AGEs amplifies the upregulation of ICAM-1 and CCL2 triggered by CD40 ligation in endothelial and Müller cells [49].

Given the increased expression of CD40 in the diabetic retina and the ability of CD40 to upregulate pro-inflammatory molecules, studies were performed to determine whether CD40 promotes development of DR. Studies in *Cd40^-/-^* mice revealed that the presence of CD40 is required for the upregulation of ICAM-1, CCL2, TNF-α, IL-1β and NOS2 as well as the development of leukostasis in the retinas of diabetic mice [41]. Diabetic *Cd40^-/-^* mice do not exhibit capillary degeneration indicating that CD40 is required for the development of experimental DR [41]. The increased expression of ICAM-1 in endothelial cells, CCL2 in Muller cells and TNF-α in microglia/macrophages in the retinas of diabetic mice required the presence of CD40 [41]. Studies in patients with DR also indicate an association between CD40 and upregulation of pro-inflammatory molecules. Retinas from patients with DR revealed co-expression of high levels of ICAM-1 and CD40 in retinal endothelial cells as well as high levels of CCL2 and CD40 in Müller cells [43].

## 6. Role of Cell Type-Specific CD40 in the Development of DR and CD40-Driven Intercellular Crosstalk in the Pathogenesis of This Disease

**Figure 2:**
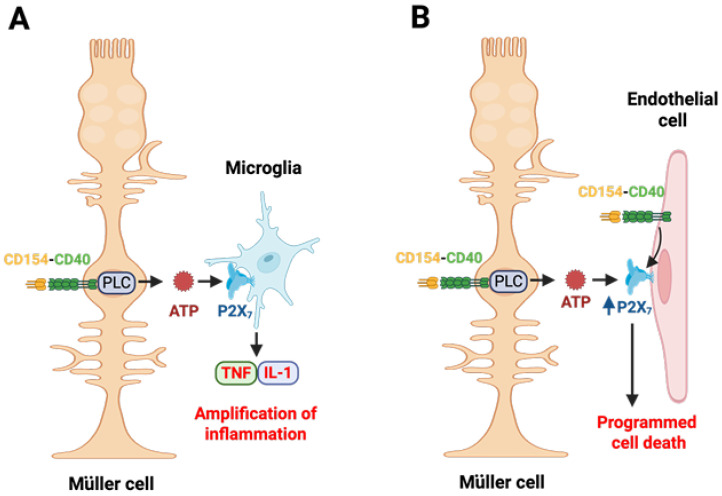
The CD40–PLCγ1–ATP–P2X_7_ signaling pathway links Müller cells with the induction of inflammatory responses in bystander microglia/macrophages and programmed cell death in retinal endothelial cells in DR. CD40 ligation in Müller cells causes PLCγ1 (PLC) activation and secretion of extracellular ATP. (**A**) P2X_7_ receptor is upregulated in microglia/macrophages in the diabetic retina. ATP binds the P2X_7_ receptor leading to secretion of TNF-α and IL-1β. (**B**) CD40 ligation in retinal endothelial cells upregulates the P2X_7_ receptor, making these cells susceptible to P2X_7_-induced programmed cell death. Created with BioRender.com.

The development of transgenic mice with cell-specific expression of CD40 further refined the role of CD40 in the regulation of inflammatory responses in the diabetic retina and the development of DR. Müller cells were chosen since these cells appear to be activated in DR and they form anatomical connections with multiple cell types in the retina [64,65]. The expression of CD40 restricted to Müller cells was sufficient to result in upregulation of CCL2, ICAM-1, TNF-α, IL-1β, NOS2, as well as the development of leukostasis and capillary degeneration in the retina of diabetic mice [57]. However, while increased CCL2 expression was detected in Müller cells from diabetic animals and Müller cells secreted CCL2 in vitro in response to CD40 stimulation, TNF-α was not detected in Müller cells from diabetic transgenic mice and primary Müller did not secrete TNF-α or IL-1β in response to CD40 ligation [57]. Moreover, ICAM-1 upregulation was detected in retinal endothelial cells, a phenomenon that was accompanied by leukostasis [57]. These findings indicated that Müller cell CD40 triggered intercellular crosstalk that resulted in the development of events key for the development of DR. These observations led to the discovery that Müller cells induce TNF-α and IL-1β expression in by-stander myeloid cells. CD40 stimulation of Müller cells caused a rapid release of extracellular ATP mediated by Phospholipase C γ1 (PLCγ1) [57]. These findings are significant since extracellular ATP can act as a messenger that triggers cytokine production by macrophages/microglia by binding purinergic receptors expressed in these cells [66]. Indeed, extracellular ATP triggered production of TNF-α and IL-1β in by-stander myeloid cells [57]. Moreover, experiments in which CD154-activated mouse Müller cells were incubated with macrophages from either wild-type mice or *P2x_7_^-/-^* animals revealed that the production of TNF-α and IL-1β in by-stander myeloid cells was dependent on the purinergic receptor P2X_7_ [57] (Figure 2A). This mechanism is operative in vivo since, in the setting of diabetes, the presence of CD40 restricted to Müller cells was accompanied by PLCγ1 activation in these cells and P2X_7_ upregulation in microglia/macrophages, a marker of activated P2X_7_ signaling [57]. Moreover, administration of the P2X_7_ inhibitor BBG markedly reduced the upregulation of TNF-α, IL-1β as well NOS2 and ICAM-1, pro-inflammatory molecules likely to be upregulated by TNF-α and IL-1β, and *P2x_7_^-/-^* mice exhibited impaired upregulation of TNF-α, IL-1β, ICAM-1, and NOS2 [57]. These studies uncovered that the expression of CD40 enables Müller cells to amplify the inflammatory responses in the diabetic retina by recruiting microglia/macrophages to secrete pro-inflammatory cytokines through purinergic signaling. Studies in patients with DR support that the CD40–PLCγ1 pathway may regulate pro-inflammatory cytokine expression in myeloid cells in patients with DR. Müller cells from these patients co-expressed activated PLCγ1 and areas of increased expression of CD40 [43]. While TNF-α was not detected in Müller cells, it was detectable in cells that appeared to be microglia/macrophages [43]. 

Besides promoting inflammatory responses in the diabetic retina, the CD40–ATP–P2X_7_ pathway also triggers programmed cell death of retinal endothelial cells [67]. CD40 is required for the development of capillary degeneration [41], a process dependent on programmed cell death of retinal endothelial cells. However, ligation of CD40 in endothelial cells does not cause cell death since CD40 can directly activate PI3K/Akt-mediated pro-survival signals [67]. The ATP–P2X_7_ pathway provides an explanation for this apparent discrepancy. P2X_7_ can cause programmed cell death through a variety of mechanisms that may include activation of caspase-1, -3 and -8 leading to apoptosis, necroptosis and activation of NLRP3 resulting in pyroptosis [68]. CD40 ligation in endothelial cells upregulates P2X_7_ expression making these cells susceptible to programmed cell death [67]. Indeed, in the presence of CD40-activated Müller cells, the release of extracellular ATP from these cells caused P2X_7_-mediated programmed cell death of retinal endothelial cells [67] (Figure 2B). The CD40–P2X_7_-programmed cell death pathway is likely operative in the diabetic retina since P2X_7_ is upregulated in retinal endothelial cells of diabetic mice, an effect that requires the presence of CD40, and the increase in programmed cell death in retinal endothelial cells in these animals is dependent on the presence of CD40 [67]. Taken together the discovery of the CD40–ATP–P2X_7_ pathway provides an explanation for how Müller cells amplify inflammatory responses in the diabetic retina by recruiting microglia/macrophages and promote the death of retinal endothelial cells. However, not all pro-inflammatory responses induced by CD40 are PLCγ1-dependent. CD40 upregulates CCL2 secretion and ICAM-1 expression in Müller cells through a mechanism that does not require PLCγ1 but is dependent on MAPK signaling [69].

CD40 signaling at the level of other retinal cells may contribute to the inflammatory response in DR. CD40 stimulation of microglia/macrophages triggers upregulation of pro-inflammatory cytokines and NOS2 [70,71,72]. Although not formally examined thus far, the upregulation of CD40 in these cells in the setting of diabetes suggest that CD40 signaling in these cells directly triggers inflammatory responses in the diabetic retina. CD40 can be induced in retinal pigment epithelial cells [44,45]. Whether CD40 ligation in these cells contributes to the pathogenesis of DR is unknown. While photoreceptors lack CD40 expression, early studies on the expression of in the retina raised the possibility that, in addition to ganglion cells, there may be a subset(s) of retinal neurons that express CD40 [42]. The relevance in DR of CD40 expressed in retinal neurons remains unexplored.

CD40 plays a pathogenic role in DR not only by regulating crosstalk among retina cells but likely also by interacting with other important mediators of DR. For example, there is crosstalk between CD40 signaling and TNF-α/IL-1β since CD40 induces production of TNF-α and IL-1β and these cytokines can upregulate CD40. AGEs upregulate CD40 suggesting that CD40 contributes to the pro-inflammatory responses induced by AGEs. CD40 activates the sensors of the unfolded protein response (UPR, see below), raising the possibility that CD40 and activated UPR sensors converge to induce pro-inflammatory responses in the diabetic retina. The likely interconnections between CD40 and other central drivers of DR may explain why inhibition of CD40 signaling is sufficient to prevent the development of DR despite the presence of multiple mediators of this disease.

## 7. VEGF and DR

VEGF is central to the pathogenesis of DR. VEGF promotes disruption of tight junctions among retinal endothelial cells leading to vascular leakage [73]. This process would result in diabetic macular edema (DME), a leading cause of vision loss in diabetics. VEGF is also a key mediator of retinal neovascularization, the central feature of PDR [73]. Neovascularization can result in vitreal hemorrhage and tractional retinal detachment, other important causes of vision loss in diabetics [74,75]. VEGF is produced by Müller cells, endothelial cells, pericytes, retinal pigment epithelial cells, and ganglion cells [74,76]. Several factors contribute to upregulation of VEGF in DR including hypoxia, the unfolded protein response (UPR, ER stress), oxidative stress, PKC activation, and advanced glycation end-products [77,78,79,80]. The central role of VEGF in the development of DME and PDR and the increased expression of VEGF in patients with DR made administration of anti-VEGF agents the leading therapeutic approach in patients with DR [81].

## 8. CD40 Promotes VEGF Upregulation in DR

CD40 increases VEGF secretion in Müller cells, a major source of VEGF in DR [46,63,82]. Given that UPR is linked to VEGF upregulation [77,83,84,85], studies examined whether CD40 ligation induces UPR activation and whether activation of UPR sensors promotes VEGF production by Müller cells. Although UPR sensors may become activated selectively depending on the stimuli [86,87], CD40 ligation in human and rodent Müller cells activated the three sensors of UPR-PERK, IRE1α and ATF6α [82]. The three sensors worked together to promote VEGF expression induced by CD40 since VEGF secretion was inhibited by knockdown of any of the UPR sensors [82]. These findings are consistent with evidence that the coordinated activation of the three UPR branches can optimally upregulate VEGF expression [88].

**Figure 3:**
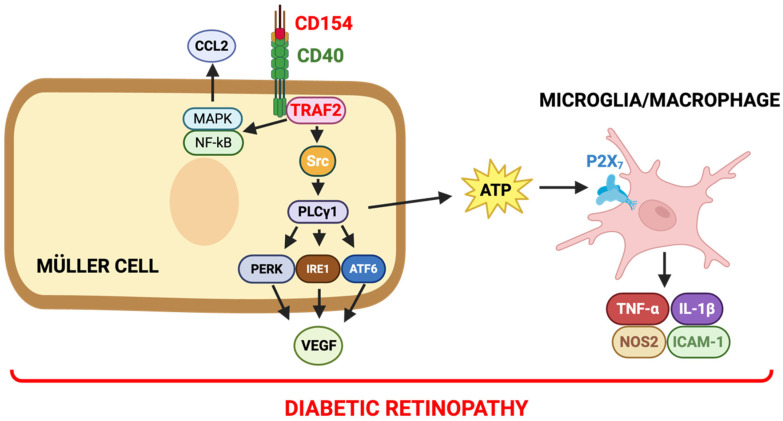
The CD40–TRAF2,3–PLCγ1 signaling pathway activates UPR sensors in Müller cells and stimulates expression of VEGF as well as upregulation of pro-inflammatory cytokines in DR. CD40–TRAF2,3 signaling is critical for activation of PLCγ1 and activation of the UPR sensors PERK, IRE1α and ATF6α. Activation of the three sensors is required for CD40-driven VEGF secretion. CD40–TRAF2,3 signaling also triggers PLCγ1-dependent activation of the ATP–P2X_7_ pathway resulting in pro-inflammatory cytokine production in microglia/macrophages. CD40–TRAF2,3 signaling activates MAPK and NF-κB resulting upregulation of CCL2 and ICAM-1 in Müller cells in a PLCY1-independent manner. Created with BioRender.com.

UPR sensors can become activated by stimuli that do not cause accumulation of misfolded proteins. Indeed, CD40 activates the three UPR branches through PLCγ1 [82], a signaling molecule that activates UPR by causing calcium flux from the ER into the cytoplasm [89]. The demonstration that PLCγ1 mediates VEGF production induced by CD40 stimulation in Müller cells indicates that the CD40–PLCγ1 pathway is responsible for two events critical for the pathogenesis of DR: upregulation of pro-inflammatory molecules in microglia/macrophages [57] and VEGF [82] (Figure 3).

Studies in mice further support the relevance of CD40 as a mediator of UPR activation and VEGF upregulation in the diabetic retina. Whereas diabetic C57BL/6 mice exhibited evidence of activation of PLCγ1, PERK, IRE1α and ATF6α as well as VEGF upregulation that partially associated with Müller cell markers, none of these changes were observed in diabetic *Cd40^-/-^* animals [82]. Activation of UPR and upregulation of VEGF were restored in diabetic *Cd40^-/-^* mice rescued to express CD40 in Müller cells [82]. Importantly, the CD40–PLCγ1–UPR–VEGF pathway is likely relevant in humans since retinas from patients with DR exhibited upregulation of CD40 in Müller cells that was accompanied by co-expression of activated PLCγ1, activated UPR sensors and VEGF [82].

CD40 ligation induces VEGF expression in endothelial cells and promotes angiogenesis in vivo [90,91]. CD40 may promote neovascularization in PDR since CD40 stimulation increases proliferation of retinal endothelial cells and VEGF expression in these cells [46]. In addition, the positive correlation between the levels of CD40 and the levels of VEGF in the vitreous of patients with PDR further suggests that CD40 plays a pathogenic role in retinal angiogenesis in these patients [46].

## 9. Role of CD40–TRAF2,3 Signaling in the Development of Retinal Inflammation, VEGF Upregulation and Development of DR

Identification of the signaling pathways downstream of CD40 that promote pro-inflammatory molecule upregulation, PLCγ1 activation, and VEGF upregulation may lead to the development of therapeutic approaches against DR based on pharmacologic inhibition of CD40 signaling; CD40 signals via TNF receptor associated factors (TRAF) [63,92,93,94]. CD40 has distal sites that recruit TRAF2 and TRAF3 and a proximal site that recruits TRAF6 [95,96]. TRAF3 competes with TRAF2, thus inhibiting CD40 signaling [97]. The result of approaches that prevent TRAF2/TRAF3 recruitment is inhibition of TRAF2 signaling [63,94]. Therefore, the recruitment of TRAF2 and TRAF6 are the key events that mediate the effects of CD40. Studies in primary retinal endothelial cells and Müller cells that express either WT CD40 or a CD40 mutant that does not recruit TRAF2,3 (CD40 ΔT2,3) [98,99] revealed that disruption of TRAF2,3 signaling caused 70–90% reduction in CD40-dependent upregulation of ICAM-1, CCL2, PGE_2_ and VEGF, as well as impaired activation of PLCγ1 [63,69]. Disruption of CD40–TRAF6 signaling also impaired these cellular responses [63]. However, in contrast to CD40–TRAF6 signaling, CD40–TRAF2,3 signaling is not required for responses key for protection against intracellular pathogens [92,93,94,100,101,102]. Thus, subsequent studies focused on the effects of genetic disruption of CD40–TRAF2,3 signaling. 

Studies using transgenic mice with expression of either WT CD40 or CD40 ΔT2,3 restricted to Müller cells revealed that diabetic mice that express CD40 ΔT2,3 in these cells did not develop upregulation of *Icam-1* and *Ccl2*, *Tnf-α*, *Il-1β*, *Nos2* and *P2x_7_* mRNA levels [69]. In addition, expression of CD40 ΔT2,3 in Müller cells impaired leukostasis, activation of PLCγ1 in Müller cells, and the development of DR [69]. Finally, disruption of CD40–TRAF2,3 signaling also impaired activation of UPR sensors and upregulation of VEGF [82]. Taken together, CD40–TRAF2,3 signaling is a central upstream driver of key pathogenic responses in DR. 

The role of TRAF2 as a mediator of the effects of CD40 likely applies to patients with DR. Areas of CD40 upregulation co-expressed high levels of TRAF2 in retinal endothelial and Müller cells in patients with DR [43]. This supports that CD40–TRAF2 signaling is activated in patients with DR since CD40 signaling can upregulate TRAF2 and the expression of TRAF2 is increased in CD40-driven inflammatory disorders [103,104,105]. Moreover, the areas of increased CD40–TRAF2 expression were associated with increased expression of ICAM-1, CCL2, activated PLCγ1, activated PERK and IRE1α as well as VEGF upregulation suggesting that the CD40–TRAF2 pathway promotes upregulation of pro-inflammatory molecules and VEGF as well as UPR activation in patients with DR [43,82].

## 10. Effects of Pharmacologic Inhibition of CD40–TRAF2,3 Signaling in DR

CD40 is a therapeutic target against numerous inflammatory/autoimmune disorders [106]. Clinical trials for the treatment of these disorders are based on administration of neutralizing antibodies against CD40 or CD154. Unfortunately, antibody-based biologics have significant drawbacks including development of infections and anti-drug antibodies that interfere with their activity. A therapeutic approach that selectively impairs CD40–TRAF2,3 signaling is expected to inhibit key responses that promote DR and circumvent drawbacks associated with the administration of neutralizing antibodies. While inhibition of CD40–TRAF2,3 may not improve abnormalities in the electroretinogram (ERG) given that diabetic *Cd40^-/-^* mice do not appear to be protected from reduction in the ERG b-wave [107], CD40–TRAF2,3 inhibition is expected to reduce retinal inflammation and VEGF upregulation, responses key for the development of DR. Moreover, administration of anti-VEGF agents in patients with DR indicate that improvement in visual acuity can be achieved without inducing changes in the ERG [108].

**Figure 4:**
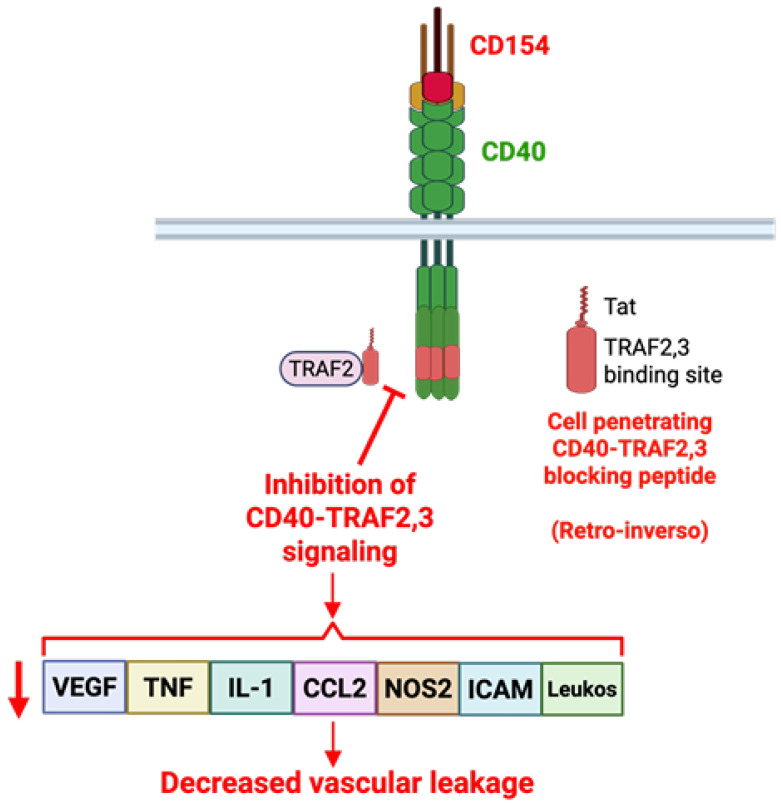
A retro-inverso cell-penetrating CD40–TRAF2,3 blocking peptide diminishes VEGF and pro-inflammatory molecule upregulation as well as vascular leakage in DR. The cell-penetrating CD40–TRAF2,3 blocking peptide consists of the amino acids from the major TRAF2,3 binding site of CD40 fused with HIV Tat_47-57_ to make it cell-permeable. It has a retro-inverso phenotype made with D-amino acids in reverse sequence. The blocking peptide would bind to TRAF2 inhibiting CD40–TRAF2,3 signaling. The blocking peptide impairs upregulation of VEGF, TNF-α, IL-1β, CCL2, NOS2, ICAM-1, leukostasis, and vascular leakage in the retinas of diabetic mice. Created with BioRender.com.

Cell-penetrating peptides represent a promising advance in ocular drug delivery [109,110]. They contain a membrane transduction domain that enables them to cross cellular membranes without interacting with specific receptors [109,110]. A 23-amino-acid CD40–TRAF2,3 cell-penetrating blocking peptide was developed as a novel approach to inhibit CD40 [63,94,111]. It contains the amino acid sequence of the major TRAF2,3 binding site in CD40, a sequence that is specific for TRAF2 and shared between humans and mice. The peptide is fused with HIV Tat_47-57_ to make it cell-permeable and has retro-inverso features making it resistant to peptidases [63,94]. It blocks CD40–TRAF2,3 signaling but not CD40–TRAF6 signaling [63,94]. Moreover, it does not inhibit CD40–TRAF6 signaling and does not impair protection against a pathogen [111]. The blocking peptide translocates into retinal cells, including Müller cells, following intravitreal injection [111]. A single intravitreal injection of the blocking peptide in diabetic mice at a time when they are expected to exhibit retinal pro-inflammatory molecule upregulation and leukostasis (2 months of diabetes) markedly impaired upregulation of *P2x_7_*, *Tnf-α*, *Il-1β*, *Nos2*, *Icam-1* and *Ccl2* mRNA as well as leukostasis assessed 2 weeks post-injection [69]. Similarly, the blocking peptide markedly impaired activation of PERK, IRE1α and ATF6α as well as upregulation VEGF in the retinas of diabetic mice [82]. Importantly, the blocking peptide drastically reduced retinal vascular leakage [82]. Thus, pharmacologic inhibition of CD40–TRAF2,3 impaired upregulation of pro-inflammatory molecules and VEGF, major drivers of the dysfunction of the blood–retinal barrier in diabetes, and as a result effectively controlled vascular leakage in the diabetic retina (Figure 4). 

## 11. Potential Therapeutic Applications of Inhibition of CD40–TRAF2,3 Signaling

Macular edema, a leading cause of blindness in diabetics [112], is caused by vascular leakage driven in part by VEGF [113,114]. Indeed, intravitreal injections of anti-VEGF agents elicit a suboptimal response in approximately 50% of patients with diabetic macular edema [36,113,114,115]. The persistently elevated expression of pro-inflammatory molecules in patients that fail anti-VEGF therapy would explain macular edema resistant to anti-VEGF agents [116]. The evidence that disruption of CD40–TRAF2,3 signaling results in marked reduction in VEGF and pro-inflammatory molecules expression supports that pharmacologic inhibition of this pathway may represent an improved approach for the treatment of DR. Moreover, the evidence that CD154 causes retinal vascular leakage after intravitreal injection in mice [46] and the increased vitreal levels of CD154 in patients with DR occur in a manner that is statistically independently of VEGF [53] suggest that targeting the CD40–CD154 pathway may have beneficial effects in DR that go beyond those related to inhibition of VEGF expression. While the studies in transgenic mice identified the effects of genetic disruption of CD40–TRAF2,3 signaling in specific cells, the likelihood that CD40–TRAF2,3 signaling acts simultaneously in various cell types to promote the development of DR support an approach to inhibit this signaling pathway in various cell types, such as the use of a cell-penetrating CD40–TRAF2,3 blocking peptide. The demonstration that the CD40–TRAF2,3 blocking peptide has long-lasting protective effects against DR may lead to novel and potentially improved treatment of this disease.

## Data Availability

No new data were created or analyzed in this study. Data sharing is not applicable to this article.
